# Function meets fabric: wearable biosensors for effortless health interaction

**DOI:** 10.1093/nsr/nwaf264

**Published:** 2025-07-22

**Authors:** Hao Bai

**Affiliations:** State Key Laboratory of Chemical Engineering and Low-carbon Technology, College of Chemical and Biological Engineering, Zhejiang University, China; Institute of Zhejiang University-Quzhou, China; ZJU-Hangzhou Global Scientific and Technological Innovation Center, China

Diagnostic and preventive healthcare services are experiencing sustained and accelerating growth across the global population, yet centralized, clinic-based models have proved inadequate in supporting timely, continuous and accessible care. Wearable technologies, especially smart textiles, provide a promising route to decentralize health monitoring by integrating biosensing capabilities directly into soft, breathable and comfortable fabrics [1]. Among various physiological signals, sweat provides a uniquely accessible and non-invasive source of molecular-level information, readily capturable at the skin–textile interface. A key obstacle to realizing this potential lies in the difficulty of obtaining sufficient sweat for reliable analysis, particularly in sedentary or low-activity scenarios. Although strategies involving hydrogels or hydrophilic fillers have been explored to better exploit natural perspiration, the sweat often remains elusive [2,3]. Active stimulation of sweat glands via iontophoresis offers a controllable alternative, yet conventional systems typically require relatively high external electric fields, which may cause skin irritation and compromise user compliance [4].

**Figure 1.**
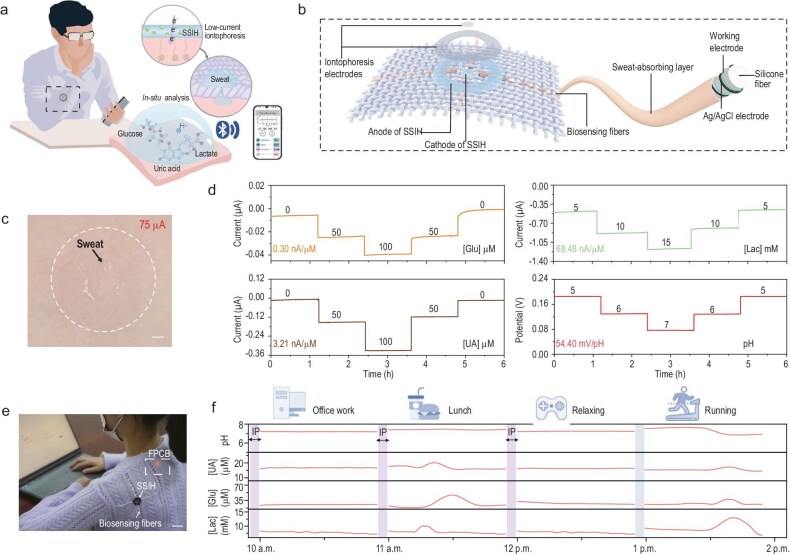
(a) Schematic overview of the integrated wearable system for simultaneous sweat induction and sensing. (b) Integrated fabric architecture combining iontophoresis electrodes with biosensing fiber array. (c) Optical image of sweat secretion induced under 75 μA iontophoretic stimulation. Scale bar, 2.5 mm. (d) Long-term responses of biosensing fibers under stepwise concentration changes. (e) Photograph of a subject wearing the integrated fabric biosensing system embedded in a sleeve during a sedentary task. Scale bar, 2 cm. FPCB, flexible printed circuit board. (f) Time-course plots of *in situ* sweat biomarker monitoring across different daily scenarios, with three iontophoretic sweat induction events. Reproduced with permission from [5].

In recent research published in the *National Science Review*, Professor Sisi He and co-workers make important contributions to wearable chemical sensing fabric that overcomes these hurdles by enabling on-demand sweat induction and stable, long-term multiplexed biomarker analysis (Fig. 1a and b) [5]. The central innovation of their system lies in the design of the sweat-induction unit, which is enabled by a novel skin-interface stabilized iontophoretic hydrogel (SSIH) electrode. This electrode exhibits superior mechanical compliance and adhesion, allowing it to form a conformal and low-impedance interface with the skin. Such intimate contact significantly enhances the efficiency of transdermal delivery for the sweat-inducing agonist. As a result, the device achieves effective sweat induction with an operational current of 75 μA (Fig. 1c), a minimal level of stimulation that is virtually imperceptible to the user. This configuration effectively decouples sweat generation from physical exertion, enabling scenario-independent, on-demand physiological monitoring.

Once perspiration is drawn out, the device transitions to its analytical role. A critical challenge in wearable sensing is the limited operational stability of conventional enzymatic sensors, which often suffer from degradation of the transducer layer in biofluids. To solve this, the authors engineered a stabilized transduction layer by depositing a protective film of nickel hexacyanoferrate over the primary Prussian blue mediator. This robust bilayer architecture effectively stabilizes the electron transduction from the enzymatic reaction to the conductive substrate, preventing signal drift and enabling continuous monitoring of multiple biomarkers for up to 6 h (Fig. 1d). The integration of these innovations into a complete wearable system was demonstrated through comprehensive on-body testing across various daily activities (Fig. 1e and f).

In summary, He and co-workers provide a first demonstration of integrating on-demand sweat induction into wearable chemical sensing fabric, with the potential to transform fields as diverse as chronic disease management and telemedicine. It would be promising to see follow-up work focusing on clinical validation in diverse populations and the expansion of the biomarker panel, further solidifying the technology's role in the future of distributed and preventative healthcare.
